# Genetic Factors in Rasmussen’s Encephalitis Characterized by Whole-Exome Sequencing

**DOI:** 10.3389/fnins.2021.744429

**Published:** 2021-10-05

**Authors:** Junhong Ai, Yisong Wang, Dong Liu, Dongying Fan, Qiqi Wang, Tianfu Li, Guoming Luan, Peigang Wang, Jing An

**Affiliations:** ^1^Department of Microbiology, School of Basic Medical Science, Capital Medical University, Beijing, China; ^2^Department of Neurosurgery, Sanbo Brain Hospital, Capital Medical University, Beijing, China; ^3^Department of Neurology, Beijing Tiantan Hospital, Capital Medical University, Beijing, China; ^4^Center of Epilepsy, Beijing Institute for Brain Disorders, Beijing, China

**Keywords:** Rasmussen’s encephalitis (RE), seizure, genetic factors, whole-exome sequencing, singe nucleotide variants

## Abstract

Rasmussen’s encephalitis (RE) is a rare chronic neurological disorder characterized by unihemispheric brain atrophy and epileptic seizures. The mechanisms of RE are complex. Adaptive immunity, innate immunity and viral infection are all involved in the development of RE. However, there are few studies on the role of genetic factors in the mechanisms of RE. Thus, the objective of this study was to reveal the genetic factors in the mechanisms of RE. Whole-exome sequencing (WES) was performed in 15 RE patients. Ten patients with temporal lobe epilepsy (TLE), which is a common and frequently intractable seizure disorder, were used as the controls. Thirty-one non-silent single nucleotide variants (SNVs) affecting 16 genes were identified in the RE cases. The functions of the genes with SNVs were associated with antigen presentation, antiviral infection, epilepsy, schizophrenia and nerve cell regeneration. Genetic factors of RE were found first in this study. These results suggest that RE patients have congenital abnormalities in adaptive immunity and are susceptible to some harmful factors, which lead to polygenic abnormal disease.

## Introduction

Rasmussen encephalitis (RE) is a rare chronic neurological disorder characterized by unihemispheric inflammation, refractory seizures, and progressive neurological deficits (Varadkar et al., 2014). It was first reported by the neurosurgeon Theodore Rasmussen in 1958 (Rasmussen et al., 1958). An epidemiological study from Germany estimated that the annual incidence of RE was 2.4 cases/10^7^ persons aged ≤ 8 years (Bien et al., 2013). The disease mainly affects children, with an average age of onset of 6–7 years (Andermann and Farrell, 2006). However, there have also been reports that adolescents and adults account for 10% of all RE cases (Villani et al., 2006). Histopathological analysis of brain specimens revealed inflammation with microglial nodules, perivascular and parenchymal infiltration of T cells, loss of neurons and astrocytes, and gliosis in the affected hemisphere (Farrell et al., 1995; Bien et al., 2005). Immunomodulatory therapy seems to slow the progression of RE but cannot stop it or change the eventual outcome (Rasmussen et al., 1958). Cerebral hemispherectomy is still the only treatment for associated seizures, but it is related to inevitable dysfunction impairment (Kossoff et al., 2003).

There are various mechanisms related to RE. Adaptive immunity, innate immunity and viral infection are all involved in the development of RE. Immunopathological studies have shown that cytotoxic CD8^+^ T lymphocytes are the most common T lymphocyte subgroup in the brains of patients with RE (Pardo et al., 2014). The intensity of peripheral CD8^+^ T cell expansion is related to the severity of the disease (Schneider-Hohendorf et al., 2016). Although the adaptive immune response is an important effector of central nervous system (CNS) damage, the innate immune response mediated by the activation of microglia and astrocytes is also the core of the pathogenesis of RE. The degree of microglial activation follows the progression pattern of RE, parallels the degree of T lymphocyte infiltration, and is observed in the early stages of cortical involvement (Pardo et al., 2004; Troscher et al., 2019).

The pathological changes observed in RE brains are similar to those in viral encephalitis, which has promoted the study of pathogen infection in the damaged cerebral cortex. Human cytomegalovirus (HCMV) and Epstein-Barr virus (EBV) infections were originally proposed by Theodore Rasmussen (Walter and Renella, 1989; Farrell et al., 1991). Other viruses were also found in RE brain samples, such as herpes simplex virus (Vinters et al., 1993; Atkins et al., 1995). The localized and slowly progressing viral infection may explain the changes in monohemispheric encephalitis in RE. However, human herpesvirus infections are very common in humans. In all these studies, the causal relationship between viruses and RE pathogenesis has not been proven.

While RE is a rare chronic brain disorder, there are few studies on the role of genetic factors in the mechanism of RE. Early reports about genetic tests in RE came from case reports and found mutations in the *NOD2/CARD15* and *SCN1A* genes (Goyal et al., 2007; Ohmori et al., 2008). Subsequently, researchers found SNPs in the *CTLA4* and *PDCD1* genes and elucidated genetic predisposition in immunoregulatory genes (Takahashi et al., 2013). However, because of the small case number or limited number of genes detected, limitations existed for the above studies. To reveal the genetic factors in the mechanisms of RE, whole-exome sequencing (WES) in 15 RE patients was performed in this study. As a result, single nucleotide variants (SNVs) were found in genes with functions in antigen presentation, antiviral infection, epilepsy, schizophrenia and nerve cell regeneration.

## Materials and Methods

### Rasmussen’s Encephalitis Patients and Controls

Between 2008 and 2016, 34 patients with RE were admitted to Sanbo Brain Hospital (Beijing, China). Clinical diagnosis was made according to the “European consensus statement” (Bien et al., 2005). Among them, 15 patients were selected and enrolled in this study. Each patient was subjected to magnetic resonance imaging (MRI) examination using an MRI scanner (Siemens 3.0T TIM Trio MRI; Munich, Germany) for diagnosis before undergoing craniotomy.

Temporal lobe epilepsy (TLE) is a common refractory epilepsy in adolescents and adults (Tellez-Zenteno et al., 2012). There were partial similarities between RE and TLE in clinical manifestations and age of onset. However, their prognoses are greatly different. Cerebral hemispherectomy is the only treatment for associated seizures in RE, but it is related to inevitable dysfunction and impairment. Surgical resection of the temporal lobe has a good outcome in TLE. Thus, TLE was used as the control group in this study.

A total of 10 temporal lobe epilepsy (TLE) patients admitted to Sanbo Brain Hospital who underwent surgical treatment were used as a control group; they were generally matched with the RE group with respect to age. Patients with other nervous system diseases were excluded.

For RE and TLE patients, at least two blocks (0.5 × 0.5 × 0.5 cm each) of brain biopsies from different areas were collected for WES after the operations.

### Whole-Exome Sequencing

WES was performed by GrandOmics (Beijing, China). Generally, the brain tissue collected during the operation was homogenized using a tissue disruptor. Then, genomic DNA was extracted with a TIANamp Genomic DNA Kit (DP304) (TIANGEN, China). DNA samples were quantified using a NanoDrop 2000. An exome-captured sequencing library was produced from the xGen Exome Research Panel v1.0 kit. The libraries were subsequently sequenced on an Illumina HiSeq X-ten sequencing instrument according to the manufacturer’s instructions with a read depth greater than 100X, and more than 95% of the area covered more than 20X.

After sequencing, the original data were obtained in FASTQ format, and then the short reads were compared with Genome Reference Consortium Human genome build 37 (GRCh37) by Burrows-Wheeler Aligner (BWA) software (v0.7–6a) (Li and Durbin, 2009) to determine the position of the short sequence on the genome. Sequence Alignment/Map (SAM) tools software (v0.1.18) was used to align these short sequences and convert the data format. The removal of redundant information and noise generated in the process of sequencing was conducted by Picard software (v1.91). Genome Analysis Toolkit (GATK) software (v2.6–4) (McKenna et al., 2010) was used to analyze sample sequencing data and the difference loci (SNPs and InDels) of the referred genome, while Annovar software (2013Aug23 version) was used for functional annotation of these mutation sites.

### Variant Filters

**FIGURE 1:**
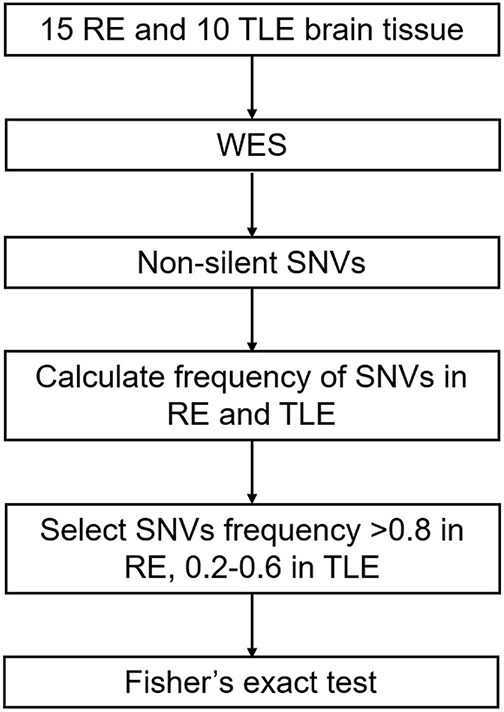
Screening process of SNVs in RE. RE, Rasmussen’s Encephalitis. TLE, temporal lobe epilepsy. WES, whole-exome sequencing. SNVs, single nucleotide variants.

We considered only canonical transcripts for each variant, assuming the most deleterious predicted effect for each transcript according to VEP software. In gene-based analyses, only non-silent SNVs were considered (that is, transcript ablations, splice donor/acceptors, splice region, stop gain, frameshift, stop lost, initiator codon variants, transcript amplifications, in-frame insertion/deletions and missense) (Figure 1).

### Statistical Analysis

The frequencies of selected non-silent SNVs in all RE and TLE cases were calculated. SNVs that had a high frequency in RE cases (> 0.8) and low frequency in TLE and normal populations (0.2–0.6) were considered to be associated with the pathogenesis of RE (Figure 1).

Fisher’s exact test was used for statistical analysis by SPSS 19.0 software. *P*-values less than 0.05 were considered to be significant.

## Results

### Clinical Features of Patients With Rasmussen’s Encephalitis

There were six males and nine females in the RE group, with a mean age of seizure onset of 7.7 years and a mean age at surgery of 11.0 years. The 10 TLE patients in the control group were generally matched with the RE patient group. There were four males and six females with a mean age of seizure onset of 7.3 years and a mean age at surgery of 9.4 years.

### Single Nucleotide Variants in Rasmussen’s Encephalitis

**FIGURE 2:**
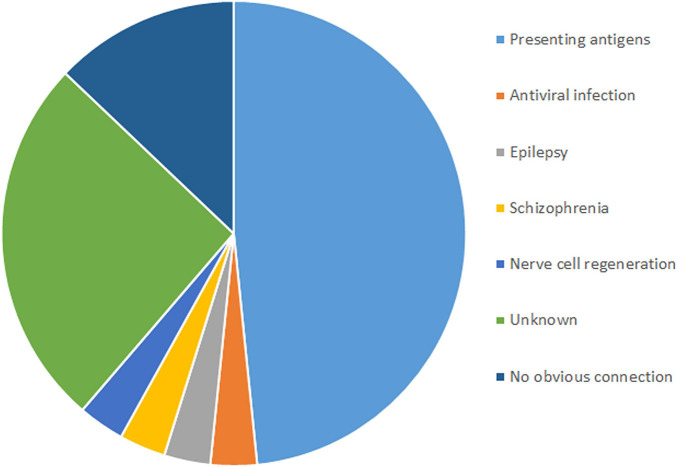
Functions and numbers of SNVs associated with RE.

In total, 31 non-silent SNVs affecting 16 genes were identified in this study (Figure 2). Detailed information on all genes and mutations is shown in Table 1. Most of the SNVs (29/31) had a frequency of 0.2–0.6 in normal populations, while two in the *MUC2* gene had a low frequency (<0.1). No rare SNVs (<0.01) associated with specific functions were found. The frequency of these SNVs in the TLE group was nearly the same as that in the normal population. However, almost all of them were carried in the RE group, with a minimum frequency of 0.8.

**TABLE 1:** Single nucleotide variants obtained by WES in 15 RE cases and 10 TLE cases.

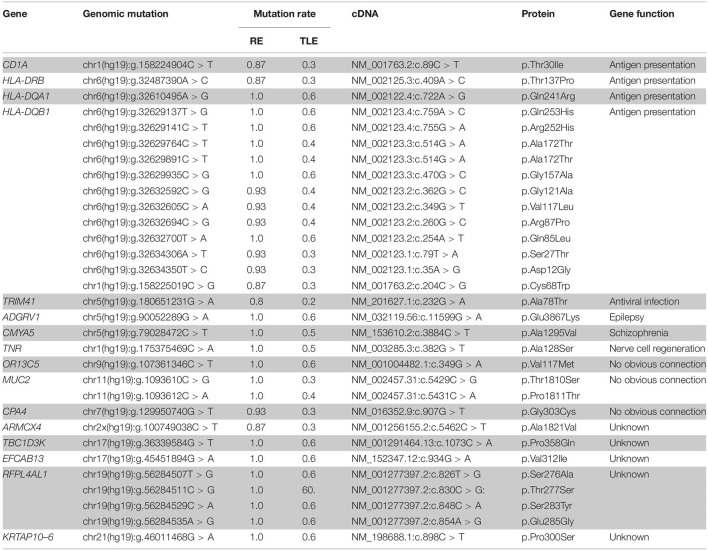

*RE, Rasmussen’s encephalitis; TLE, temporal lobe epilepsy; cDNA, complementary DNA.*

**FIGURE 3:**
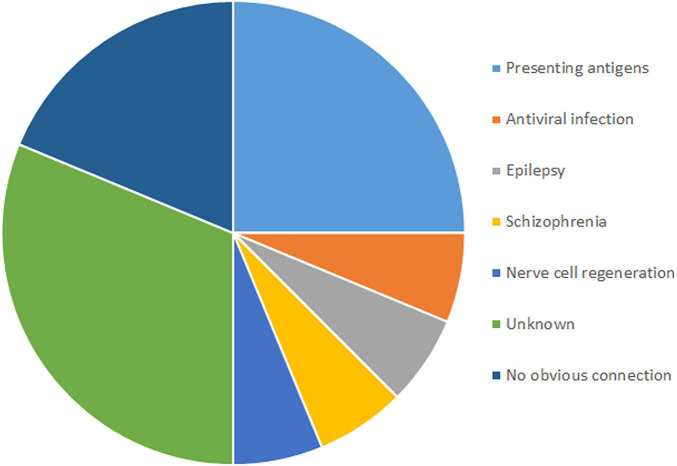
Functions and numbers of genes carried SNVs associated with RE.

Among the 16 genes with SNVs, half were related to antigen presentation, antiviral infection, epilepsy, schizophrenia and nerve cell regeneration, five had unknown functions, and three had no obvious connection with RE (Figure 3).

### Genes With Single Nucleotide Variants in Rasmussen’s Encephalitis Are Related to Antigen Presentation and Antiviral Infection

SNVs in four genes related to antigen presentation and one gene related to antiviral infection were found in RE cases (Figure 3). Genes related to antigen presentation were *HLA-DQA1, HLA-DQB1, HLA-DRB5*, and *CD1A*. The gene related to antiviral infection was *TRIM41*.

HLA-DQA1, HLA-DQB1, and HLA-DRB5 belong to HLA class II. More than 11 SNVs were found in the *HLA-DQB1* gene, while one SNV was found in the *HLA-DQA1* and *HLA-DRB5* genes. One SNV was found in *CD1A*. In addition, there was one SNV in *TRIM41* gene in RE.

### Genes With Single Nucleotide Variants in Rasmussen’s Encephalitis Are Related to Epilepsy, Schizophrenia and Nerve Cell Regeneration

Three genes related to epilepsy, schizophrenia and nerve cell regeneration were found in RE in this study. *ADGRV1* plays a role in epilepsy. *CMYA5* was reported to have a connection to schizophrenia. *TNR* is related to nerve cell regeneration.

One SNV was found in the *ADGRV1, CMYA5*, and *TNR* genes in RE cases.

## Discussion

The mechanisms of RE are complex. Viral infection was originally proposed to be the possible cause of RE (Walter and Renella, 1989; Farrell et al., 1991). Over the years, several studies have reported the detection of a number of viruses, including enteroviruses, EBV, human HCMV, human papillomavirus and herpes simplex virus (HSV), in the brains of RE patients (Pardo et al., 2014). Furthermore, in our previous research, antigens of various human herpesviruses (HHVs), including HCMV, EBV and HHV6, were detected in RE brain tissues with positive rates ranging from 50 to 88.5% (Zhang et al., 2017; Liu et al., 2018). In addition, it was found that the interferon-induced transmembrane protein-3 (IFITM3) polymorphism rs12252-C is associated with the high detection rate of HCMV and rapid disease progression in RE patients with the IFITM3 rs12252-CC genotype. This result indicates that IFITM3 rs12252-C is related to the disease progression of RE patients by promoting persistent HCMV infection in brain tissue (Wang et al., 2021).

Drug-resistant epilepsy is one of the most important clinical features of RE (Bien et al., 2002; Pradeep et al., 2016). TLE is a common and frequently intractable seizure disorder. Both RE and TLE have complex pathogenesis and show similar syndromes. Thus, TLE was used as the control group in this study. Studies have shown that adaptive and innate immunity play an important role in the mechanisms of RE (Varadkar et al., 2014; Schneider-Hohendorf et al., 2016). However, the triggering factor of these immune responses remains unknown. In this study, WES was used to explore the genetic factors of RE. The results showed that SNVs related to antigen presentation and antiviral infection were found in RE cases. However, none of them were found in TLE cases. This suggested that unlike TLE patients, RE patients might have congenital abnormalities in adaptive immunity. Therefore, we speculated that the triggering factors of the adaptive immune response in brain tissue in RE cases may be SNVs related to antigen presentation and antiviral infection when facing the challenge of viral infection.

In addition to adaptive immune-related genes, SNVs were also found in genes involved in epilepsy, schizophrenia and nerve cell regeneration in RE brain tissue but not in TLE tissues. This result suggested that SNVs in genes with the function of epilepsy, schizophrenia and nerve cell regeneration may be responsible for neurological symptoms in RE cases. Although RE and TLE have similar clinical manifestations, the prognosis of them after surgical treatment is completely different. The outcome for most patients with TLE is good. However, the prognosis of RE is not satisfactory and is accompanied by progressive neurological dysfunction. Based on the results of this study, we speculate that it may be due to mutations in genes related to nerve cell regeneration in patients with RE, which inhibited the recovery of nervous system function. Simultaneously, due to mutations in genes related to antigen presentation and antiviral infection, the patient’s adaptive immune function is dysregulated, and the antiviral effect is weakened, which in turn leads to aggravation of neurological diseases.

RE mainly affects children and is a pediatric epileptic syndrome in this population. Genetic factors and neurodevelopmental abnormalities were reported to be associated with age-specific epileptic syndromes, such as infantile spasms (IS). According to reports, approximately 7∼8% of infants with IS with unknown etiology have abnormal copy number variation (Osborne et al., 2010; Mefford et al., 2011). However, studies about the role of genetic factors in RE are lacking.

In this study, genetic factors of RE were explored. SNVs were found in genes with the functions of antigen presentation, antiviral infection, epilepsy, schizophrenia and nerve cell regeneration. We speculated that congenital abnormalities in adaptive immunity in RE would be the basis of neuron lesions, causing neurologic symptoms such as progressive neurological and cognitive deterioration and unihemispheric brain atrophy. Furthermore, SNVs in genes with functions in epilepsy, schizophrenia and nerve cell regeneration in RE cases may increase the degree of epilepsy.

In summary, genetic factors of RE were found in this study. We speculated that the triggering factors of the adaptive immune response against brain tissue in RE cases may be SNVs related to antigen presentation and antiviral infection when facing the challenge of viral infection. Congenital abnormalities in adaptive immunity increase neuropathy damage. Our results not only contribute to further understanding of the complex pathogenesis of RE but also help further elucidate the etiology of the disease. In the future, a large number of RE samples and *in vitro* experiments are needed to verify the roles of these SNPs in the pathogenesis of RE.

## Data Availability Statement

The datasets presented in this study can be found in online repositories. The names of the repository/repositories and accession number(s) can be found below: NCBI SRA BioProject, accession no: PRJNA761444.

## Ethics Statement

The studies involving human participants were reviewed and approved by the Ethics Committee of Sanbo Brain Hospital, Capital Medical University. Written informed consent to participate in this study was provided by the participants’ legal guardian/next of kin.

## Author Contributions

JAi analyzed the data and wrote the manuscript. YW and DL collected data of the patients. DF contributed reagents and materials. TL and QW participated in analysis and discussion of the results. GL, PW, and JAn conceived and designed the experiments and the revision of the manuscript. All authors read and approve the final manuscript.

## Conflict of Interest

The authors declare that the research was conducted in the absence of any commercial or financial relationships that could be construed as a potential conflict of interest.

## Publisher’s Note

All claims expressed in this article are solely those of the authors and do not necessarily represent those of their affiliated organizations, or those of the publisher, the editors and the reviewers. Any product that may be evaluated in this article, or claim that may be made by its manufacturer, is not guaranteed or endorsed by the publisher.
